# Leukocyte Mitochondrial DNA Copy Number in Blood Is Not Associated with Major Depressive Disorder in Young Adults

**DOI:** 10.1371/journal.pone.0096869

**Published:** 2014-05-08

**Authors:** Ying He, Jinsong Tang, Zongchang Li, Hong Li, Yanhui Liao, Yanqing Tang, Liwen Tan, Jindong Chen, Kun Xia, Xiaogang Chen

**Affiliations:** 1 Institute of Mental Health, the Second Xiangya Hospital of Central South University, Changsha, Hunan, China; 2 Institute of Genomic Medicine, Wenzhou Medical University, Wenzhou, Zhejiang, China; 3 Department of Psychiatry, First Affiliated Hospital, China Medical University, Shenyang, Liaoning, China; 4 The State Key Laboratory of Medical Genetics, Central South University, Changsha, Hunan, China; 5 Key Laboratory of Psychiatry and Mental Health of Hunan Province, Central South University, Changsha, Hunan, China; 6 The State Key Laboratory of Medical Genetics, Central South University, Changsha, Hunan, China; Kunming Institute of Zoology, Chinese Academy of Sciences, China

## Abstract

**Background:**

Major depressive disorder (MDD) is the leading cause of disability worldwide, and has significant genetic predisposition. Mitochondria may have a role in MDD and so mitochondrial DNA (mtDNA) has been suggested as a possible biomarker for this disease. We aimed to test whether the mtDNA copy number of peripheral blood leukocytes is related to MDD in young adults.

**Methods:**

A case-control study was conducted with 210 MDD patients and 217 healthy controls (HC). The mtDNA copy number was measured by quantitative polymerase chain reaction (qPCR) method. Depression severity was assessed by the Hamilton-17 Depression Rating Scale (HDRS-17).

**Results:**

We found no significant differences in mtDNA copy number between MDD patients and HC, though the power analysis showed that our sample size has enough power to detect the difference. There were also no significant correlations between mtDNA copy number and the clinical characteristics (such as age, age of onset, episodes, Hamilton Depression Rating Scale (HDRS) score and Global Assessment of Function Scale (GAF) score) in MDD patients.

**Conclusion:**

Our study suggests that leukocyte mtDNA copy number is unlikely to contribute to MDD, but it doesn’t mean that we can exclude the possibility of involvement of mitochondria in the disease. Further studies are required to elucidate whether mtDNA can be a biomarker of MDD.

## Introduction

Major depressive disorder (MDD) is a severe and complicated mental illness that causes considerable impairment, which has a 12-month prevalence of 6.6% and a lifetime prevalence of 16.2% [1]. The World Health Organization has predicted MDD to be the second leading cause of disability worldwide by the year 2020 [2].

Though the etiology of the disease remains unclear, even environmental circumstances have proven to influence the aetiology of the disease, genetic factor is still believed to have an important role in its pathogenesis. Twin studies suggest a heritability of 40% to 50%, and family studies indicate a twofold to threefold increase in lifetime risk of developing MDD among first-degree relatives [3]. Due to the complex phenotype and the unrepeatable results, the progress to find common genetic variant in MDD has been frustrated. But there were some intriguing findings as described below.

MDD is a brain disease characterized by persistent depressed mood or loss of interest or pleasure from daily activities, and brain is highly dependent on mitochondria’s energy production. That’s why a "mitochondrial psychiatry" model of depression has been proposed [4], so the relationship between MDD and mitochondria has been explored in several studies as follows. In the first place, depression symptoms have been described in patients with kinds of mitochondrial disorders such as external ophthalmoplegia [5], MELAS (mitochondrial encephalomyopathy, lactic acidosis and stroke-like episodes) [6], [7] and oxidative phosphorylation disease [8]. The comorbidity facts which are most likely, at least in part, a consequence of overlapping neural mechanisms, gave us a clue that mitochondrial dysfunction might be a possible etiopathogenesis of MDD. Second of all, post-mortem studies found reduction of products linked to mitochondrial gene expression in frontal cortex [9] and cerebellum [10] samples from depressed patients. While not only human, rats subjected to depression model were found the inhibition of mitochondrial respiratory chain in cerebral cortex and cerebellum [11]. Furthermore, using the magnetic resonance sounding (MRS) [12], [13] and positron emission tomography (PET) [14] technique, abnormalities of high-energy phosphate metabolism were found in the brain of subjects with MDD. Besides the muscle biopsy took a more direct approach, showing the decrease of mitochondrial adenosine triphosphate production rate (MAPR) and enzyme ratio in the MDD patient in comparison with controls [15]. In addition, patients with depressive disorder were reported to have more mitochondrial DNA (mtDNA) deletion mutations than controls [15], [16] or share the same mtDNA mutation with mitochondrial diseases [5], [17]–[19]. Last but not least, there were some studies that show “matrilineal inheritance” in some depression affected families. For example, depression was reported at very high prevalence in the probable maternal inheritance mothers [18] and a modest maternal bias in the susceptibility towards the development of depression was found [20]. Combined with the fact that mtDNA is inherited exactly through the maternal lineage, we hypothesized that mtDNA may be involved in the pathogenesis of MDD.

So far, most depression studies focus on the point mutation of mtDNA as well as the mitochondrial function, but there have been few studies about the relationship between depression and mtDNA copy number which represents the mtDNA content is important because the mtDNA copy number as a surrogate measure of mitochondrial integrity was supposed to regulate mitochondrial function [21], [22]. Kim et al. had reported that low leukocyte mtDNA copy number is related to depression in community dwelling old women [23]. However, in a recent study, de Sousa et al. found no difference in mtDNA copy number between young adults with bipolar depression and healthy controls [24]. Furthermore, though MDD can develop at any age, the peak ages at onset of MDD are between 20 and 25 years as well as between 40 and 45 years, yet those late onset cases are often associated with structured brain abnormalities like cerebral vascular lesions. Given this, we designed the following case-control study to determine whether leukocyte mtDNA copy number is associated with MDD in young adults.

## Materials and Methods

### Subjects

The complete details of the entire study design and procedures involved were in accordance with the Declaration of Helsinki. Risks and benefits of the study were presented in detail, and all participants gave written informed consent. If participants were mentally or legally incapable of giving consent, such as minors, their guardians were contacted to fill out the written consent form on the patients' behalf. The study was approved by the ethics committee of the Second Xiangya Hospital of Central South University.

This study included 210 MDD patients which were sought through Outpatient Department, the Second Xiangya Hospital (100 male and 110 female, aged 17–45 years, mean age = 30.2±8.10 years) and 217 matched healthy controls (HC) (109 male and 108 female, aged 18–45, mean age = 30.8±7.05 years). The patients and HC recruited in this study were biologically unrelated and all were of the Han Chinese ethnicity. All patients were 17–45 years old and diagnosed strictly according to the DSM-IV-TR (Diagnostic and Statistical Manual of Mental Disorders, Fourth Edition, Text Revision) [25] criteria by a board-certified psychiatrist. Inclusion criteria for HC were: male or female 17–45 years of age, not meeting DSM-IV-TR criteria for an axis I disorder and not having a family history of psychiatric disorder. Exclusion criteria for all subjects included: active substance abuse; serious organic disorders (such as stroke, heart failure and uremia) and other hereditary diseases.

### Depression and function assessment

MDD patients were questioned on general parameters, including age, gender, age of onset, marital status, profession, level of education, medication and number of previous episodes. Depression severity was assessed with the Hamilton-17 Depression Rating Scale (HDRS-17) [26]. Psychological, social, and occupational function was assessed with Global Assessment of Function Scale (GAF), which is a 100-point scale described in the DSM-IV-TR [25] on page 34.

### Measurement of leukocyte mtDNA copy number

Peripheral leukocyte DNA from 500 µL of whole blood sample was extracted using the TIANamp Blood DNA Kit DP318 (Tiangen Biotech, Beijing, China) and stored at −80°C avoiding repeated freeze-thaw cycles. DNA was checked for purity and concentration, using the NanoDrop 2000 Spectrophotometer (Thermo Scientific, Wilmington, DE, USA), and all had OD_260_/OD_280_ values of 1.8–2.0. The relative mtDNA copy number was normalized to a single-copy nuclear β-globin gene and simultaneously measured by quantitative polymerase chain reaction (qPCR) and the 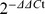 method [27], [28].

Forward and reverse primers of β-globin were: 5’-GCTTCTGACACAACTGTGTTCACTAGC-3’ and 5’-CACCAACTTCATCCACGTTCACC-3’, respectively and forward and reverse primers of mtDNA were: 5’-CACCAGCCTAACCAGATTTC-3’ and 5’-GGGTTGTATTGATGAGATTAGT-3’, respectively [29], [30]. Amplification and detection were conducted in 96-well plates with CFX96 Touch real-time PCR detection system (BioRad Laboratories, Hercules, CA, USA). The PCR reaction mix consisted of 10 ng of DNA mixed with 5 µL Maxima SYBR Green qPCR Master Mix (Thermo Fisher Scientific, Waltham, MA, USA) and 4 pmol of each primer in a final volume of 10 µL. The PCR condition were as follows: initial denaturation at 95°C for 10 minutes; amplification by using 35 cycles including denaturation at 95°C for 15 s, annealing at 60°C for 30 s, and extension at 72°C for 30 s. All samples were run in triplicate and only the average Ct values were determined. The mtDNA copy number was calculated with the following equation: relative copy number = 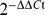 (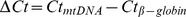)

### Statistical analysis

Statistical analyses were conducted with SPSS 17.0. Group results are presented as mean ± S.D. The usual threshold of significance (p) was fixed at 0.05 for two-tailed test. Levene’s test was used to confirm the homogeneity of variances between the groups. Relationships between variables were evaluated with the Spearman’s rank correlation test. Power analysis was performed using the G*Power (version 3.1.7) (http://www.psycho.uni-duesseldorf.de/abteilungen/aap/gpower3)

## Results

Both groups were well matched for age and gender (for general characteristics of the recruited subjects see Table 1). Patients had mean age of onset of 28.8 (±8.31) and 122 (58.1%) had medication history.

**Table 1:** General characteristics of the recruited subjects.

Variable	MDD (n = 210)	HC (n = 217)
**Age, years (mean ± S.D.)**	30.2±8.10	30.8±7.05
**Gender (male, female)**	100M, 110F	109M, 108F
**Age of onset, years (mean ± S.D.)**	28.8±8.31	
**Medication, n (%)**	122 (58.1)	
**First-episode, n (%)**	127 (60.5)	
**HDRS score (mean ± S.D.)** *	21.0±7.76	
**GAF score (mean ± S.D.)** **	58.0±10.03	

MDD: major depressive disorder; HC: healthy controls; M: male; F: female; HDRS: Hamilton Depression Rating Scale; GAF: Global Assessment of Function Scale.

^*^ HDRS were to rate the severity of depression in MDD patients, the higher the score, the more severe the depression.

^**^ GAF assigns a clinical judgment in numerical fashion to the MDD patients’ overall functioning level. The scale ranges from 0 (inadequate information) to 100 (superior functioning).

**Figure 1:**
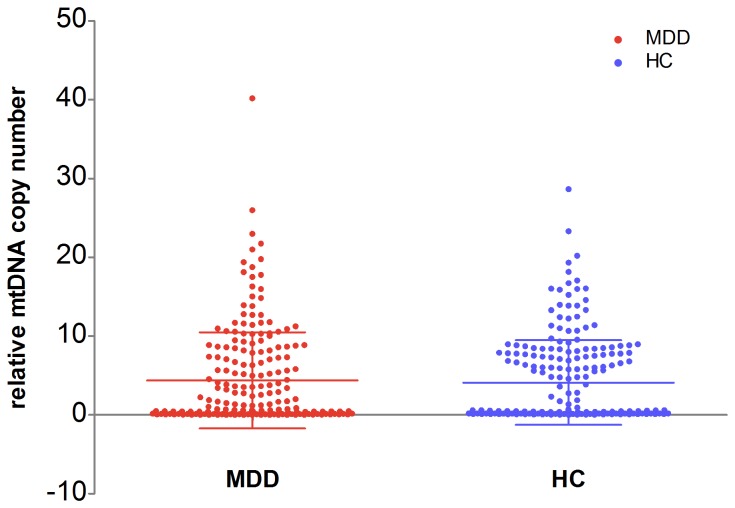
Relative mtDNA copy number of MDD patients and HC. There is no significant difference in mtDNA copy number between MDD and HC.

The case-control analyses did not show any significant differences in mtDNA copy number between MDD and HC (MDD patients = 4.4±6.10; healthy controls = 4.1±5.38) (p = 0.650) (see Figure 1), even when gender were considered (p = 0.228 and p = 0.621 for males and females respectively). Then we separated the MDD patients into two groups respectively according to episodes, medication and HDRS score, still did not see any statistically significant differences amongst two groups for either of the variables concerned (see Table 2).

**Table 2:** Comparison of mtDNA copy number between the MDD group and the HC group, and comparison of mtDMA copy number within the MDD group.

Variable	mtDNA copy number (mean ± S.D.)		P-value
	MDD (n = 210)	HC (n = 217)	
**Sex**			
Male	4.9±6.07	4.0±5.24	0.228
Female	3.9±6.11	4.3±5.55	0.621
P-value	0.218	0.684	
**Episodes**			
First episode	3.8±5.38		
Recurrence	5.2±7.01		
P-value	0.116		
**Medication**			
Take medicine	4.9±6.63		
Did not take medicine	3.6±5.21		
P-value	0.120		
**HDRS score** *			
score≥18	3.9±5.37		
score<18	5.5±7.39		
P-value	0.107		

MDD: major depressive disorder; HC: healthy controls; HDRS: Hamilton Depression Rating Scale.

p calculated by the t-test.

^*^ Patients are considered to be currently suffered from depression with a score of ≥18 on the HDRS, while are defined as being in remission if they had an HDRS score less than 18.

As mtDNA copy number did not show a normal distribution, the relationships between variables were evaluated with Spearman’s rank correlation test and were considered as very weak to negligible correlations when the absolute values of the correlation coefficients (r_s_) were less than 0.2. We found some very weak positive correlations between mtDNA copy number, episodes and GAF score, also some very weak negative correlations between mtDNA copy number, age, age of onset, education and HDRS scores. But none of these correlations met statistical significance (for details see Table 3).

**Table 3:** Correlations between mtDNA copy number and other variables in MDD patients.

Variables	r_s_	P-value
**Age**	−0.080	0.251
**Age of onset**	−0.098	0.157
**Episodes**	0.095	0.168
**Education**	−0.042	0.548
**HDRS score**	−0.145	0.035*
**GAF score**	0.004	0.956

r_s_: Spearman's rank correlation coefficient; HDRS: Hamilton Depression Rating Scale; GAF: Global Assessment of Function Scale.

p calculated by the Spearman’s rank correlation test.

^*^ p = 0.035<0.05, but the correlation coefficient (r_s_) = −0.145>−0.2, so the correlation between mtDNA copy number and HDRS score are still not significant.

Based on the data of the previously published study in depression [23], we could calculate out the effect size is 0.41. Then, we used this effect size to calculate the power of the current study. The results show that our sample has reasonable power under various effect size value (99% for exactly 0.41, 92% for 0.41*80%, 72% for 0.41*60%), which means our sample size is enough to detect the difference in leukocyte mtDNA copy number between these two groups.

## Discussion

Mitochondria are cytoplasmic organelles of the eukaryotic system that play central roles in energy metabolism, free radical production, calcium homeostasis and apoptosis [31]. mtDNA is vital for maintaining normal mitochondrial function and energy production for the body [32]. mtDNA copy number which appears to have high heritability is very important for normal mitochondrial function [33]. Altered mtDNA copy number regulation can result in diseases such as multiple sclerosis [34], renal cell carcinoma [33], type 2 diabetes [35], cardiomyopathy [36], breast cancer [37] and also suggested to be associated with aging [38]. So we wondered that when depression occurred in brain which is an energy intensive organ, how leukocyte mtDNA copy number would change.

The results of our study suggest that no significant leukocyte mtDNA copy number variation distinguishes the MDD and HC. Moreover, leukocyte mtDNA copy number also did not show a significant difference related age, age of onset, education, medication situation, episodes, HDRS score and GAF score. That means, to some extent, no matter how old is the patient, no matter if he/she is taking antidepressant and whether he/she is currently suffered from depression or being in remission, leukocyte mtDNA copy number is not notably affected.

Our finding is inconsistent with Kim et al.’s finding of a positive association between low leukocyte mtDNA copy number and depression in old women [23], but is consistent with the results of a recent study in young adults with bipolar depression [24]. This may due to the different age span that both our subjects are much younger than the old women evaluated by Kim et al. And there is a potential explanation of our results is the “threshold hypothesis of mtDNA copy number control” which means there are some thresholds which work to push the mtDNA copy number towards a middle-range [39]. Thus, we postulate that the depression symptoms might not be severe enough to break the threshold and cause mtDNA copy number decline. Combining with the positive result of mtDNA copy number in old depressive women and our negative result in young adults, we postulate that as people grew older the compensation ability weakened, threshold down-regulated, finally lead to a decreasing in mtDNA copy number.

However the result from this study should be carefully interpreted because it has a numbers of limitations. Relative mtDNA copy number is not accurate enough, could have missed some information. We did not evaluate mitochondrial function to test whether it has a relationship with mtDNA copy number and MDD. Furthermore the mtDNA copy number varies among different tissues [40], but we don’t know the standard leukocyte mtDNA copy number and have no idea about whether leukocyte mtDNA can represent the whole body’s mitochondrial activity or the brain’s metabolic level.

## Conclusion

Based on our analysis in this study, we conclude that leukocyte mtDNA copy number is unlikely to contribute to MDD. Despite the negative findings, it is still meaningful as the first study showing the relationship between leukocyte mtDNA copy number and MDD in young adults. But it also doesn’t mean we can exclude the possibility that mitochondria may be involved in the development of MDD. On the contrary, our study indirectly reflects that mtDNA mutation may play a more important role in mtDNA expression or mitochondrial dysfunction and even in MDD. For this reason, further studies are required to elucidate if mtDNA is associated with MDD, determine how it influence the progress of MDD and whether mtDNA can be a biomarker of MDD.
